# Development of a surgical safety checklist for the performance of radical nephrectomy and tumor thrombectomy

**DOI:** 10.1186/1754-9493-6-27

**Published:** 2012-12-15

**Authors:** Shivam Joshi, Michael A Gorin, Rajinikanth Ayyathurai, Gaetano Ciancio

**Affiliations:** 1 Miami Transplant Institute, Miami, FL, USA; 2 James Buchanan Brady Urological Institute, Johns Hopkins Medical Institutions, Baltimore, MD, USA; 3 Department of Urology, University of Miami Miller School of Medicine, Miami, FL, USA; 4 Jackson Memorial Hospital, Miami, FL, USA; 5 Department of Surgery, Division of Transplantation, University of Miami Miller School of Medicine, Post Office Box 012440, Miami, FL, 33101, USA

**Keywords:** Checklist, Renal cell carcinoma, Inferior vena cava, Tumor thrombus

## Abstract

**Background:**

The surgical management of renal cell carcinoma with invasion of the renal vein or inferior vena cava is associated with significant rates of perioperative morbidity and mortality. In this report we propose a surgical checklist aimed at reducing adverse events associated with the resection of these tumors.

**Methods:**

This review describes the development of an evidence- and experience-based surgical checklist aimed at improving the perioperative safety of patients undergoing radical nephrectomy and tumor thrombectomy.

**Results:**

Reducing the risk of complications during the surgical management of renal tumors with venous invasion begins with appropriate pre-operative imaging aimed at defining the cranial extent of the tumor thrombus, thus facilitating accurate preoperative planning. Other key elements of the checklist are aimed at ensuring clear and precise pre-, intra- and postoperative communication between members of the multidisciplinary-care team.

**Conclusion:**

A standardized surgical checklist may help to increase the perioperative safety of patients undergoing radical nephrectomy and tumor thrombectomy. Future validation studies are required to determine the clinical feasibility and post-implementation safety profile of this new checklist.

## Introduction

Renal cell carcinoma (RCC) is known to invade the renal vein or inferior vena cava (IVC) in 4 to 10% of cases [1]. The management of these tumors is limited to surgical intervention which is associated with significant rates of perioperative morbidity and mortality ranging from 11.5% to 25% and 1.5% to 6%, respectively [2-4]. The high rate of complications associated with radical nephrectomy and tumor thrombectomy has drawn attention to ways to improve the safety of this procedure.

**Table 1:** Checklist for the Performance of Radical Nephrectomy and Tumor Thrombectomy

**Preoperative Workup and Surgical Planning**				
History and physical exam performed	Yes		No	
Exam findings suggestive of a lack of collateral venous flow	Yes		No	
Medical consultations obtained and recommendations acted on	Yes		No	
Preoperative labs reviewed and abnormalities addressed	Yes		No	
MRI or CT scan within last 30 days	Yes		No	
Level of thrombus	I	II	III	IV
Presence of bland thrombus on imaging	Yes		No	
Complete IVC obstruction on imaging	Yes		No	
Presence of venous collaterals on imaging	Yes		No	
Ancillary surgical teams consulted	Yes		No	
Need for an IVC filter to be placed preoperatively	Yes		No	
Informed consent obtained	Yes		No	
Anticoagulation therapy addressed	Yes		No	
**Day of Surgery/Operating Room**				
Ancillary teams reminded	Yes		No	
ICU team notified	Yes		No	
Medications and allergies re-reviewed	Yes		No	
Previous anesthesia history reviewed	Yes		No	
Airway and aspiration risk evaluated	Yes		No	
Labs obtained day of surgery reviewed	Yes		No	
Surgical Site Marked	Yes		No	
Blood products available	Yes		No	
Cell saver available	Yes		No	
Display of appropriate imaging	Yes		No	
Anticipated equipment sterilized and in the room	Yes		No	
Introduction of all team members	Yes		No	
Confirm patient identity, procedure and site	Yes		No	
Delivery of antibiotic prophylaxis	Yes		No	
Arterial, peripheral, and central line placement	Yes		No	
TEE available	Yes		No	
Need for VVP and/or CPBP assessed and available	Yes		No	
Need for IVC resection assessed and graft material available	Yes		No	
**Case Closure and Sign-out**				
Instrument, sharp, and towel counts correct	Yes		No	
Surgical specimens marked and identified	Yes		No	
Brief operative note completed	Yes		No	
Patient presented to ICU/recovery team notified	Yes		No	

Borrowing from lessons learned in the aviation industry, in 2008 the World Health Organization (WHO) unveiled a universal surgical safety checklist aimed at minimizing perioperative morbidity and mortality [5]. Only one year after its debut, the checklist was shown to be effective in significantly improving surgical safety [6]. The lead author of the WHO checklist has previously described the keys to a successful checklist, which include: brevity, clarity, and usability [7].

In the field of urology, surgical checklists have recently been described for the performance of transurethral resection of bladder tumor as well as partial nephrectomy [8,9]. Herein, we propose a checklist for performing radical nephrectomy for RCC with renal vein or IVC involvement. Our checklist is divided into three sections: (1) preoperative workup and surgical planning (2) the operating room, and (3) case closure/sign-out.

## Methods

A safety checklist for the surgical management of RCC with tumor thrombus was developed based on a review of the English-language literature and our own operative experience (Table 1). More specifically, PubMed was searched for contemporary articles which describe various technical aspects of performing radical nephrectomy for tumors with venous invasion. Those articles relevant to patient safety were reviewed and common practices were incorporated into our checklist.

### Checklist components

#### Preoperative workup and surgical planning

##### Patient selection

Surgery is indicated in patients without evidence of metastatic disease who are otherwise fit for surgery. In the absence of metastatic disease, those undergoing surgery have 5-year survival rates approaching 70% [10]. Additionally, surgery may be considered in patients with metastatic disease for palliative measures such as to reduce pain or vena caval syndrome. However, the median survival for this subset of patients is estimated at less than 2 years [11].

##### Preoperative evaluation

A complete history and physical examination should be performed for all patients. Special attention should be paid to the patient’s past medical history and performance status, as many patients will require referral to a cardiologist or internist for medical optimization prior to surgery. Further, the surgeon should document the presence or absence of lower extremity edema, which is an important factor in determining whether a patient can tolerate a surgical interruption of the IVC. The absence of lower extremity edema suggests the development of venous collaterals.

Other components of the preoperative workup include routine lab work (i.e. serum electrolytes, a complete blood count, liver function tests and a coagulation panel), chest x-ray and possibly a nutritional assessment. Working in collaboration with a medicine specialist, additional studies may be required to evaluate a patient’s cardiac and respiratory status. This may include a stress test and/or an echocardiogram. In addition, cessation of preoperative anticoagulation and the need for bridging therapy should also be addressed.

##### Operative planning

The most important information needed for operative planning is the level of tumor extension. Adverse event rates have been shown to increase with higher levels of the tumor thrombus [12]. Preoperative imaging can accurately identify the level and volume of the tumor thrombus, presence of a coexisting bland (i.e. blood) thrombus, presence of contralateral disease, and if caval wall invasion has occurred.

The presence of a coexisting bland thrombus increases the possibility that the patient will need to undergo an IVC resection, or less preferably placement of an IVC filter [13]. Magnetic resonance imaging (MRI) is currently the gold standard for detecting a tumor thrombus level with 100% sensitivity [14]. In patients with metallic implants (e.g. pacemakers, metal plates or screws), MRI is typically contraindicated and multidetector computerized tomography can be used instead [15]. Preoperative imaging should ideally be performed within 14 days of surgery and at the longest within 30 days of surgery [16].

##### Anesthesia and surgical consultations

Prior to the day of surgery, the patient should be evaluated by the anesthesia team. This should include an assessment of the patient’s airway, prior anesthesia history, and medical comorbidities. In addition, the surgeon should begin communication with other surgical specialists who may be required to aid in the procedure. Commonly this includes a vascular and/or cardiothoracic surgeon. For complex cases, it is often necessary to hold a multidisciplinary meeting several days in advance of the procedure.

On the day of surgery, operative cases that may require the assistance of ancillary care teams should not start until the appropriate teams have been notified or reminded of their possible need. Patients postoperatively going to the intensive care unit should be presented to the ICU team the morning of surgery. Finally, any specific surgical items or equipment should be requested at this point to allow sufficient time for them to be acquired prior to the beginning of the surgery.

### Operating room

Since serious complications can arise even with the management of level I tumor thrombi, all patients should have an arterial, peripheral, and central line placed and also have blood products readily available. For level I and level II tumor thrombi, our group has had a median estimated blood loss of 275 mL with 75% of patients requiring at least 1 transfused unit of blood [17]. However, the quantity of blood products needed on stand-by should increase with higher level tumor thrombi. For level III tumor thrombi, the mean blood loss was 500 mL (range 100 to 5,000) [18]. As would be expected, the management of supradiaphragmatic tumor thrombi are the most complex and have had a mean blood loss of 2960 mL (range 500 to 7000) requiring a mean 9 units of blood (range 0 to 16) [19]. Cell saving techniques should be considered in patients who are expected to have massive blood loss or are unable to receive homologous blood products.

Prior to incision, a “time out” should be performed in accordance with the recommendations of the WHO [5]. Important aspects of a “time out” include introduction of all team members; confirmation of the appropriate patient, site, and procedure; the display of appropriate imaging; the delivery of antibiotic prophylaxis, and that any anticipated critical, surgical events have been reviewed. It is important for the nursing staff to ensure that all necessary surgical instruments are readily available and have been appropriately sterilized. Some aspects of the checklist may seem repetitive or mundane; however, they serve an important purpose in minimizing preventable errors. An evaluation of the success of the WHO’s checklist showed a decrease in overall death rate from 1.5% to 0.8% (*P*= 0.003) and inpatient complications from 11.0% to 7% (*P*< 0.001) in first 30 days after surgery [6].

Depending on the level of the tumor thrombus, the surgical management can be more or less complex. Our group has previously described the important steps involved in the surgical procedures for the management of different levels of tumor thrombi [17-21]. We briefly review several important aspects:

#### Venovenous bypass (VVBP)

VVBP can be used in the treatment of level III and IV tumor thrombi to avoid complications associated with decreased venous return to the heart. However, VVBP introduces its own set of risks including the possibility for lymphocele, infection, vessel injury due to vascular access, and air embolism [22,23] . VVBP also may not be needed given that a large, chronic thrombus would have fostered the development of collaterals through the lumbar, azygous, and hemiazygous veins [24].

#### Cardiopulmonary bypass (CPBP) with or without deep hypothermic circulatory arrest (DHCA)

For tumor thrombi extending above the diaphragm, the traditional approach has been the use of a sternotomy and CPBP with DHCA [25,26]. CPBP has also been used for patients undergoing removal of major tumor emboli from the pulmonary artery. However, CPBP is associated with several complications including coagulopathy, central nervous system dysfunction, increased risk of increased blood loss and transfusion requirements, perioperative renal dysfunction, and multi-organ failure [25,26]. CPBP should be reserved only for large intra-atrial tumor thrombi or major tumor emboli. CPBP can be avoided in patients with smaller atrial thrombi or those terminating at the cavo-atrial junction using an entirely transabdominal approach [18]. A transabdominal approach can be used to safely remove tumors with IVC involvement [18-21].

#### *Intraoperative* transesophageal echocardiography *(TEE)*

Intraoperative TEE is indicated for all tumors which extend to at least the level of the major hepatic veins. In these cases, TEE is essential as it provides real-time information regarding the proximal extent of the tumor which may change with manipulation of the IVC or arterial clamping.

#### IVC resection

Resection of the vena caval wall should be avoided when the tumor is free floating and therefore can be easily extirpated following a simple cavotomy. However, it may be necessary to resect the IVC in cases of adherent or invasive tumors so as to ensure complete local resection. Of note, one report showed no 5-year survivors in the setting of incomplete local resection [27]. In a recent report from our group, we observed that the presence of a bland thrombus increases the likelihood that IVC wall invasion is present requiring IVC resection [13]. In the setting of a large, long-standing thrombus, collaterals may be present and likely preclude the need for IVC replacement. Clinically, complete venous obstruction without the presence of collaterals presents as lower extremity edema and dilated abdominal wall veins. Radiographically, collaterals can be seen as dilated azygous, hemiazygous, or lumbar veins [24]. Cases lacking collateral circulation which require complete IVC resection typically necessitate the use of a synthetic interpositional graft.

#### Presence of bland thrombus

Concomitant bland thombus is present in 15-20% of cases with level II-IV tumors [13]. While some groups advocate for the preoperative placement of an IVC filter to prevent an embolic event, we disagree with this practice as it risks incorporation of the filter into the thrombus.

### Case closure and signout

Prior to closure of the surgical incision, the surgical team should ensure that the instrument, sharp, and towel counts are correct. Any surgical specimens being sent for pathological analysis should be appropriately marked and identified. A brief operative note should be completed prior to patient transport to ensure accurate communication to teams in the post-operative recovery area or ICU. At the time of patient handoff, the surgeon should speak directly with the receiving team to ensure continuity of care.

## Conclusion

Radical nephrectomy for RCC with venous invasion is associated with high rates of perioperative morbidity and mortality. The proposed surgical checklist aims to improve the perioperative safety for patients undergoing this procedure. Future validation studies are required to determine the clinical feasibility and post-implementation safety profile of the proposed checklist.

## Abbreviations

CPBP: Cardiopulmonary bypass; DHCA: Deep hypothermic circulatory arrest; ICU: Intensive care unit; IVC: Inferior vena cava; MRI: Magnetic resonance imaging; RCC: Renal cell carcinoma; TEE: Transesophageal echocardiography; VVBP: Venovenous bypass; WHO: World health organization.

## Competing interests

The authors declare that they have no competing interests.

## Authors’ contributions

GC and RA conceived the manuscript. SJ and MAG drafted the manuscript. All authors participated in the critical revision of the manuscript. All authors read and approved the final manuscript.
